# Serum Cartilage Oligomeric Matrix Protein in Late-Stage Osteoarthritis: Association with Clinical Features, Renal Function, and Cardiovascular Biomarkers

**DOI:** 10.3390/jcm9010268

**Published:** 2020-01-18

**Authors:** Jana Riegger, Martin Rehm, Gisela Büchele, Hermann Brenner, Klaus-Peter Günther, Dietrich Rothenbacher, Rolf E. Brenner

**Affiliations:** 1 Department of Orthopedics, Division for Biochemistry of Joint and Connective Tissue Diseases, University of Ulm, 89081 Ulm, Germany; jana.riegger@uni-ulm.de; 2 Institute of Epidemiology and Medical Biometry, Ulm University, Helmholtzstraße 22, 89081 Ulm, Germany; martin.rehm@uni-ulm.de (M.R.); gisela.buechele@uni-ulm.de (G.B.); dietrich.rothenbacher@uni-ulm.de (D.R.); 3 Division of Clinical Epidemiology & Aging Research, German Cancer Research Center (DKFZ), 69120 Heidelberg, Germany; h.brenner@dkfz.de; 4 Network Aging Research, University of Heidelberg, 69115 Heidelberg, Germany; 5 University Center of Orthopaedics and Traumatology, University Medicine Carl Gustav Carus Dresden, TU Dresden, 01069 Dresden, Germany; Klaus-Peter.Guenther@uniklinikum-dresden.de

**Keywords:** knee osteoarthritis, hip osteoarthritis, COMP, renal function, NT-proBNP, cystatin C, biomarker, WOMAC, FFbH

## Abstract

This study aimed to assess associations between serum cartilage oligomeric matrix protein (sCOMP) and phenotypic characteristics in late-stage hip and knee Osteoarthritis (OA) as well as its correlation with further serum markers of possible comorbidities in the Ulm Osteoarthritis Study. Moreover, the prognostic relevance of preoperative sCOMP concentrations for short-term functionality and pain outcomes after hip or knee joint replacement was explored. Preoperative serum samples and detailed information about the health status (i.e., WOMAC scores, Hannover Functionality Status (FFbH)) of 754 OA patients undergoing total joint replacement were included. Spearman rank-correlation coefficients and multiple linear regression models were used to evaluate the relationships between sCOMP, other serum markers, and health outcomes. There was a significant positive association between sCOMP and markers of renal (cystatin C, creatinine, and eGFR) and cardiac (e.g., NT-proBNP) impairment. Since renal failure might cause accumulation of sCOMP, additional adjustment with eGFR was performed. Preoperative sCOMP levels in knee OA but not hip OA patients were positively associated with FFbH, WOMAC function sub-scale and total WOMAC scale as well as the post-operative WOMAC stiffness sub-scale six months after surgery. Our data clearly demonstrate an association between sCOMP and renal function as well as other confounding factors, which should be considered in future biomarker studies.

## 1. Introduction

Osteoarthritis (OA) of hip and knee joints represents one of the most frequent causes of disability and pain worldwide [1]. Besides genetic predisposition, age, gender, obesity, preceding joint injury, as well as comorbidities such as the metabolic syndrome, are known risk factors associated with articular cartilage degeneration [2]. The increasing prevalence of OA during the last decades emphasizes the importance of more effective preventive and/or therapeutic strategies based on reliable diagnostic and prognostic markers [3]. In late-stage OA, joint replacement is mostly the last effective treatment option to alleviate pain and loss of functionality (including joint stiffness) in order to improve the quality of life [4]. While hip and knee arthroplasty are highly successful and cost-effective in most cases [5], about 10% to 20% of patients do not benefit much from this surgical approach, an aspect which is still insufficiently understood [6,7].

Since metabolic alterations of chondrocytes usually precede the structural damage of cartilage, associated biomarkers are highly relevant for early diagnosis and might also allow a prediction of rapid disease progression. Biomarkers studied in OA are mostly associated with extracellular matrix turnover of cartilage, joint inflammation, or general metabolic parameters [8,9]. Although a broad spectrum of potential systemic markers has been tested only a few of them have repeatedly shown promising results. Previous studies reported associations between the severity of the disease and urinary C-terminal cross-linked telopeptide of type II collagen (uCTX-II), serum hyaluronan, cartilage oligomeric matrix protein (COMP), and C-reactive protein (CRP) [10,11]. A recent meta-analysis highlighted that serum COMP and CTXII can distinguish knee and hip OA patients from controls at early stages, though only COMP was effective in predicting the progression of the disease [12]. 

COMP, also referred to as thrombospondin 5, is a pentameric glycoprotein, which is expressed in cartilage and numerous other tissues/cells including ligaments, synovium, fibroblasts/myofibroblasts, vascular smooth muscle cells, cardiomyocytes, and various tumor cells [13]. It is known to be involved in collagen secretion and fibrillogenesis, and interacts with various other components of the extracellular matrix (ECM) like collagen types I, II, IX, matrillin 3, aggrecan, and fibronectin [10,14]. While mutations in the COMP gene are associated with two forms of skeletal dysplasia—pseudoachondroplasia and multiple epiphyseal dysplasia—altered expression and/or systemic release of wild type COMP has been described in a number of predominantly age-dependent and degenerative diseases such as osteoarthritis, cardiomyopathy, fibrosis as well as breast and prostate cancers [13,15]. Primarily, COMP has been considered as a quite specific indicator of cartilage ECM turnover, which is elevated during joint degeneration in osteoarthritic disease or rheumatoid arthritis but also after intense loading due to excessive physical activity or joint trauma [16,17,18] but it becomes more and more clear that various tissues and diseases contribute to its serum level. 

In the late stages of OA, the elevated synovial fluid and serum levels of COMP (sCOMP) are declining probably due to its depletion in highly degenerated tissue [16,18,19]. However, locally high expression was reported in chondrocytes adjacent to severely damaged cartilage, indicating a possible implication in insufficient repair processes [20]. Nevertheless, the clinical value of sCOMP in the late stage of OA and the confounding effects of other diseases still has to be evaluated in more detail. 

Therefore, the present analysis focuses on the possible association of sCOMP concentrations with epidemiologic and phenotypic characteristics in late-stage hip and knee OA as well as its correlation with further serum markers associated with possible comorbidities in the well-characterized cohort of the Ulm Osteoarthritis Study [21]. This exploratory analysis gives an idea of specific pathogenetic pathways or phenotypes that might be associated with COMP and should be further considered, i.e., as potential confounders in osteoarthritis cohorts. In addition, a potential prognostic relevance of preoperative (baseline) sCOMP values for short-term outcomes after joint replacement, determined by the Hannover Functionality Status Questionnaire (FFbH) and the Western Ontario and McMaster University Osteoarthritis Index (WOMAC), was also investigated in an explorative manner.

## 2. Experimental Section

### 2.1. Study Population

All patients included within the longitudinal Ulm Osteoarthritis Study had advanced OA of the hip or knee and underwent the first unilateral total joint replacement between January 1995 and December 1996 in one of four large hospitals in the southwest of Germany (for further details see [21,22,23,24]). The inclusion criteria (i.e., Caucasian, age <76 years, absence of malignancies, inflammatory diseases, rheumatoid arthritis, or corticosteroid medication; no previous joint replacement) were fulfilled by overall N = 809 patients with radiographic changes of grade ≥2 according to Kellgren and Lawrence [25]. The study was approved by the local Ethics Committee of the University Ulm (No. 40/94 and 164/14) and written informed consent of the patients was given.

### 2.2. Sample and Data Collection

Information about the patients including demographic data, previous medical history, comorbidities and symptoms (e.g., diabetes mellitus type 2, hypertension, myocardial infarction, heart failure, overweight/obesity) was obtained by standardized interview and examination performed by trained physicians before surgery (=baseline) as well as six months after surgery (=follow-up). Radiologic evaluation of contralateral joints and both hands was performed to classify patients as having unilateral, bilateral, or generalized OA (the latter if at least two finger joints and at least one first carpometacarpal joint was affected in addition to the replaced hip or knee joint). Secondary OA was assumed in case of infection, avascular necrosis, osteochondritis dissecans, hemorrhagic diathesis, slipped femoral capital epiphysis, or acetabular dysplasia of the hip and preceding trauma with radiologically and/or surgically confirmed structural joint lesion [22]. Missing information about laterality, generalization, and cause of OA as well as comorbidities was handled as a separate category referred to as “unknown”. Body weight and height were used to calculate the body mass index (BMI) [26]. At baseline as well as six months after joint replacement functionality was determined by the FFbH [23], which addresses the general function during the last seven days, and the sub-scale “function” of the WOMAC osteoarthritis index [27], focusing on the affected/ operated joint at the time-point of assessment. Moreover, the WOMAC sub-scales “stiffness” and “pain” were used to evaluate these clinical outcome parameters and to calculate the total WOMAC score. 

Non-fasting serum samples were taken preoperatively by standard venipuncture and stored at −80 °C. Analysis of uric acid, aspartate aminotransferase (AST), alanine aminotransferase (ALT), alkaline phosphatase (AP), calcium, phosphate, and creatinine was performed in a central laboratory at the time of baseline recruitment according to routine methods. The glomerular filtration rate (eGFR) was estimated using the creatinine-based formula of the Chronic Kidney Disease Epidemiology Collaboration (CKD-EPI) [28]. High-sensitivity C-reactive protein (hsCRP) was measured using a commercial kit (NA-latex CRP, Behring Werke, Marburg, Germany), calibrated with the WHO reference standard 85/506 [24]. For measurements conducted in the year 2019 from frozen never thawed samples, an electrochemiluminescence immunoassay (ECLIA; Cobas Elecsys 411, Roche, Mannheim, Germany) was used to measure the following parameters: growth differentiation factor-15 (GDF-15) coefficient of variation (CV) < 5%, lower limit of detection (LOD) of 10 ng/L, measuring range 27.6–12,700 ng/L), N-terminal pro-B-type natriuretic peptide (NT-proBNP) CV < 5%, LOD of 5 pg/mL), high-sensitivity cardiac troponin T (hs-cTnT) with a LOD equal to 5 ng/L. High-sensitivity cardiac troponin I (hs-cTnI) was measured on an ARCHITECT STAT (Abbott Diagnostics), with a within-laboratory imprecision of ≤10% CV across the range of 10 to 50,000 ng/L (LOD < 2.0 ng/L), and a reported within-run and within-laboratory CV < 5.5%. Serum cystatin C (CysC) was measured by immunonephelometry on a Behring Nephelometer II (inter-assay CV 2.9–3.2%).

### 2.3. Quantification of sCOMP

Baseline sCOMP (sCOMP) was analyzed by means of a commercial sandwich ELISA against human COMP (BioVendor, Heidelberg, Germany; LOD < 0.4 ng/mL). The assay was run according to the manufacturer’s instructions, using a sample dilution of 1:50. Both inter (CV 15.6%) and intra assay variations (CV 2.8%) were assessed as quality control. The absorbance was detected at 450 nm and a reference wavelength at 630 nm by using the multimode microplate reader Infinite M200 Pro (Tecan Austria GmbH, Groedig, Austria). 

### 2.4. Statistical Analysis

Participants were categorized into four groups based on the quartiles of serum COMP levels at baseline (Quartile 1: <600.4 ng/mL; 2: 600.4–799.5 ng/mL; 3: 799.6–1068.3 ng/mL; 4: >1068.3 ng/mL). The characteristics of the patients were described using descriptive statistics. Distributions of continuous variables were presented as means (with standard deviation, SD) or medians (with first quartile, Q1, and third quartile, Q3) and those of categorical variables as the frequency with the corresponding percentage of the total cohort and within each sCOMP quartile.

Based on the skewed distribution observed in serum COMP concentrations, medians were calculated across the strata of the various covariates. Generalized linear models were used to test associations of selected socio-demographic and clinical variables with serum COMP concentration when they were controlled for eGFR or age, gender, BMI, and eGFR. For further analysis, serum COMP concentrations were natural log-transformed, which led to a nearly normal distribution. 

Partial Spearman rank correlation coefficients, adjusted for age, sex, BMI, and in addition also for eGFR, were calculated to evaluate the relationships between sCOMP and other serum marker concentrations at baseline. Associations between sCOMP and health status scores at six months follow-up (FFbH, WOMAC pain, stiffness, function, and total score) were evaluated using multiple linear regression models adjusted for age, sex, BMI, and eGFR.

Statistical analysis was performed with SAS version 9.4 (SAS Institute, Cary, NC, USA) and R version 3.5.1 (R Foundation for Statistical Computing, Vienna, Austria).

## 3. Results

Overall, sCOMP quantification could be performed for 754 patients representing 93.2% of the total cohort. Parallel preoperative sCOMP values and FFbH assessment at baseline and six months after the joint replacement were available for 77.1% and 61.7% of the overall cohort, respectively (Table A1). All WOMAC sub-scales could be evaluated from 73.2% and 54.1%, respectively (Table A2).

### 3.1. Associations between sCOMP and Other Laboratory Parameters at Baseline—With and without eGFR Adjustment

After adjustment for age, sex, and BMI, the sCOMP concentration of OA patients was positively correlated to parameters commonly associated with cardiac (NT-proBNP) and renal (cystatin C, creatinine) impairment and negatively correlated with eGFR (Table 1). Moreover, also AST, which can be considered as a marker of both liver and myocardial tissue damage, showed a positive association with sCOMP. No statistically significant correlation was found for uric acid, ALT as a primary liver marker, calcium, and phosphate, as well as further cardiac risk markers hs-cTnT and hs-cTnI. 

With respect to the statistically significant associations between sCOMP and all tested parameters of renal function (serum creatinine, cystatin C, and eGFR), an additional adjustment concerning eGFR was included. Due to this consideration, we found a negative association of sCOMP and hsCRP, while the trend for a positive association with AP and GDF-15 was diminished. However, the significant association of cystatin C with sCOMP was preserved after eGFR adjustment.

### 3.2. Associations between sCOMP and Clinical Characteristics of OA Patients at Baseline—With and without eGFR Adjustment

Increased levels of sCOMP were positively associated with age, male gender, and higher BMI (Table 2) at baseline. Further patient characteristics were consequently analyzed after adjustment for these potential confounders. Using this approach, we could not detect any significant associations between sCOMP values and the tested patient characteristics. This included the presence of hip or knee OA, bilateral OA, primary or secondary cause of OA, smoking status or the presence of patient-reported co-morbidities diabetes, hypertension, cardiac infarction, and cardiac insufficiency. Moreover, no significant association was found with regard to the radiologic Kellgren and Lawrence score in hip and knee OA patients (Table S1A,B). However, after additional adjustment for eGFR a positive trend between sCOMP and generalized OA was found.

### 3.3. Correlation between sCOMP and FFbH Functionality for Patients with Hip and Knee OA

Since patients with knee OA had higher median sCOMP values as compared to those with hip OA (Table 2) and with respect to previous studies on early stages of OA cautiously pointing to a joint-specific relevance as a biomarker [12], the subsequent evaluation of clinical scores at baseline and six months after joint replacement was performed separately for these two localizations. 

**Figure 1:**
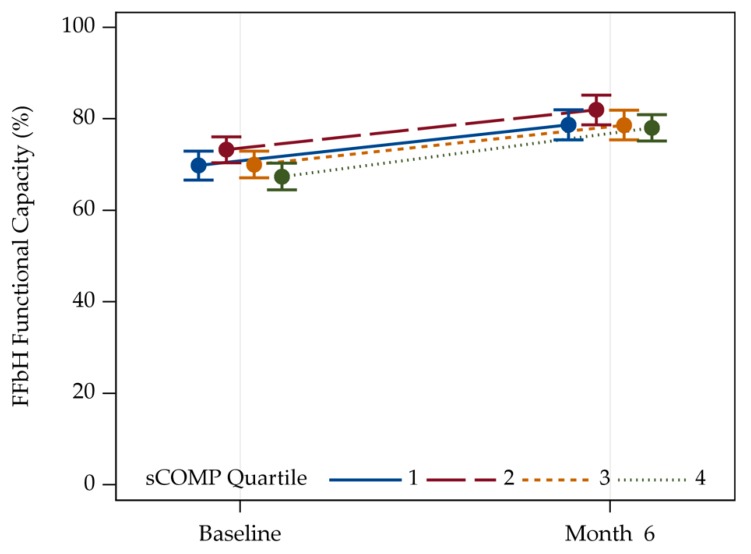
Trajectories of the FFbH Hannover Functionality Status of patients with knee OA. Values are given as means with two-sided 95% confidence limits. sCOMP Quartile 1: N = 79, Quartile 2: N = 85, Quartile 3: N = 88, Quartile 4: N = 108.

Overall, joint replacement led to an improvement in general physical function assessed by the FFbH in both hip (Figure S1) and knee OA (Figure 1) as assessed six months after surgery.

Using adjustment for age/sex/BMI and eGFR in hip OA patients, the sCOMP values were not associated with FFbH score, which is an established tool for assessing more general physical function neither at baseline nor six months after joint replacement (Table S2). In patients with knee OA, however, the FFbH score was statistically significantly associated with sCOMP at baseline but not six months after arthroplasty (Table 3). 

### 3.4. Association between sCOMP and WOMAC in Patients with Hip and Knee OA

**Figure 2:**
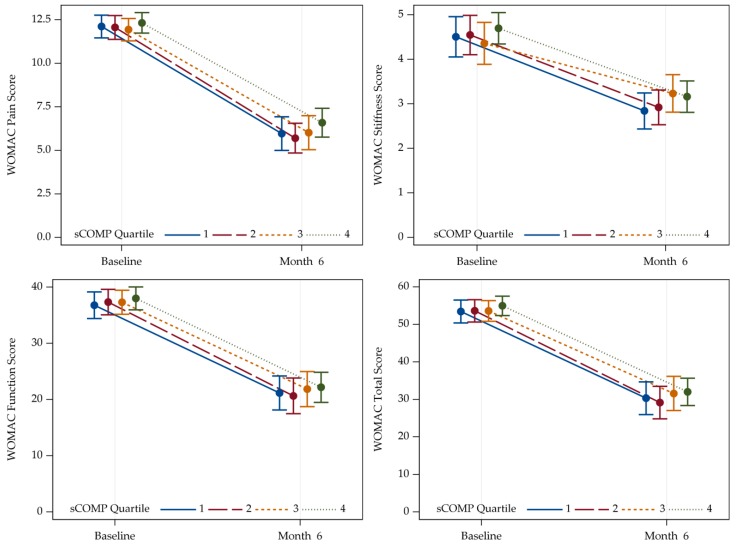
Trajectories of separated WOMAC sub-scales pain (**A**), stiffness (**B**), and function (**C**), and total WOMAC scores (**D**) of patients with knee OA at baseline and six months follow-up. Higher values reflect more severe impairment. Values are given as means with two-sided 95% confidence limits. sCOMP Quartile 1: N = 79, Quartile 2: N = 85, Quartile 3: N = 88, Quartile 4: N = 108. WOMAC = Western Ontario and McMaster University Osteoarthritis Index; Q = quartile.

In general, relevant improvement in all WOMAC sub-scales (pain, stiffness, and function) as well as in the total WOMAC score could be shown in patients with hip (Figure S2) and knee OA (Figure 2A–D) six months after surgery. 

In line with the results based on FFbH, with adjustment for age/sex/BMI and eGFR no statistically significant association of sCOMP was found for WOMAC pain, stiffness, and function sub-scales as well as the total score in case of hip OA, neither at baseline nor six months after joint replacement (Table S3A,B).

For knee OA—also in agreement with the FFbH evaluation—a positive association was detected at baseline for the sub-scale function and the total score (Table 4A). Furthermore, before knee joint replacement, a trend could be noticed for an association of sCOMP with the WOMAC pain sub-scale and sCOMP (*p* = 0.071). Six months after joint replacement there was no association with pain or function sub-scales as well as the total WOMAC score. Interestingly, however, a significant association was observed between baseline sCOMP and the WOMAC stiffness score at this follow-up time-point (Table 4B). 

## 4. Discussion

The analysis of sCOMP in the Ulm Osteoarthritis Study revealed novel aspects about the biomarker itself, its relation to phenotypic features, and comorbidities in advanced OA of the hip or knee. In particular, we found a significant correlation between sCOMP concentration and serum markers of renal impairment (cystatin C, creatinine, and eGFR), implying the consideration of appropriate adjustment of these confounders. Independent of renal function there was a positive association of sCOMP with a distinct subset of biomarkers related to cardiovascular disease including NT-ProBNP and cystatin C, although the self-reported manifest cardiovascular disease was not associated. Moreover, we described that high sCOMP levels were positively associated with the preoperative FFbH and WOMAC sub-scale function as well as the total WOMAC score in knee OA patients, but not in hip OA patients.

Fernandes et al. reported that COMP might suit as a diagnostic marker in early asymptomatic OA that did not exhibit any radiological abnormalities [29]. Similar conclusions were drawn by Verma and Dalal, who found that sCOMP was associated with pain but not radiological changes [16]. Furthermore, they described an overall negative association between the level of sCOMP and disease progression—an association which might be explained by the gradual depletion of interterritorial COMP found in human late-stage OA cartilage [19]. Nevertheless, Verma and Dalal proposed that sCOMP might serve as a prognostic marker, which allows determining patients at risk of rapid disease progression in the early stages of OA [16].

In line with previous studies, we also found a positive association with sex, age, and BMI [16] but none concerning the smoking status [30]. Although the participants of the present study all suffered from late-stage joint degeneration in the symptom-leading hip or knee joint, we found that after age/sex/BMI/eGFR-adjustment sCOMP levels of patients with generalized OA tended to be higher in comparison to patients without additional multisite hand-OA. It has been suggested that sCOMP concentration depends on the volume of the affected cartilage area, which could also explain higher sCOMP levels in male as compared to female patients [31]. According to Fernandes et al., the association between the number of OA-affected joints and the sCOMP level might also be important with regard to the overall, not only joint-specific, burden of disease, including identification of further early osteoarthritic but still asymptomatic joints that cannot be revealed by radiograph [29]. Using adjustment for age/sex/BMI w/wo eGFR, however, we did not observe a statistically significant difference of sCOMP between uni- and bilateral hip or knee OA. 

Interestingly, we observed several associations between sCOMP and other disease-related serum parameters. The strong association with markers of renal function—cystatin C, creatinine, and eGFR—suggests that the COMP serum level is not only dependent on the release from different tissues but might also be modulated by renal function and the capacity of its excretion. In agreement with these results, increased COMP levels have been previously identified in the urine of horses with aseptic joint diseases [32]. Therefore, any impairment of renal excretion would result in an accumulation of sCOMP, as reported for other substances, e.g., cardiac biomarkers [33,34]. This represents an important finding since sCOMP is currently regarded as one of the most promising and best studied OA biomarkers. However, eGFR or any other measure of renal function has not previously been considered as possible confounder. As an example of a potential relevance, only the adjustment for eGFR revealed a significant negative association between sCOMP and the inflammation marker hs-CRP, as previously described in the Rotterdam study cohort [10]. 

While hyperuricemia was previously found to be associated with generalized OA in hip OA patients within the Ulm Osteoarthritis Study [35], no significant correlation with sCOMP was detected in the present study with or without additional eGFR adjustment.

Although enhanced AST levels might suggest an association between sCOMP and hepatic disease, no association was found with respect to ALT [36]. AST can also be regarded as a marker of cardiomyocyte integrity, which matches with the positive association found between sCOMP and NT-proBNP, though no significant association between sCOMP and self-reported manifest cardiac insufficiency, hypertension, or cardiac infarction could be found after adjustment. Nevertheless, osteoarthritis is known to be associated with an enhanced risk for long-term cardiovascular diseases [37] and COMP is expressed in vascular smooth muscle cells as well as cardiomyocytes and implicated in cardiovascular pathology [38]. Moreover, sCOMP has already been described to be associated with coronary calcifications in patients with coronary heart disease [39]. It has been suggested that enhanced sCOMP levels result from the proteolytic activity of ADAMTS-7 (a disintegrin and metalloproteinase with thrombospondin motifs-7), which promotes the degradation of COMP in blood vessels and thus mediates vascular calcification and atherosclerosis [38,39,40]. 

However, high levels of COMP were found to be associated with a lower risk of incident myocardial infarction in a case–control study [41]. This observation might be related to the implication of COMP as a natural inhibitor of thrombin which could explain a respective protective functionality [42]. Interestingly, the significant association of cystatin C with sCOMP was preserved after eGFR adjustment, indicating that this association is at least partly independent of renal function. In this regard, the relevance of cystatin C as a cardiovascular risk factor even after adjustment for renal function might come to the fore [43]. An alternative explanation could be that cystatin-C is an inhibitor of cathepsin K, a cysteine proteinase known to degrade collagen type II and aggrecan, two major extracellular matrix components of cartilage. Kozawa et al. described elevated levels of cathepsin K expression in OA cartilage and a positive correlation with cystatin C expression compatible with a positive feedback mechanism [44]. Therefore, cystatin C might also represent a potential biomarker for OA itself. The troponin levels (hs-cTnT, hs-cTnI), which rather reflect myocardial damage and are also regarded as prognostic markers of chronic heart disease, were not associated with sCOMP in our cohort. Thus, the clinical relevance of the differential risk factors for chronic cardiac disease in osteoarthritis clearly deserves further investigation. Nevertheless, our results indicate that sCOMP levels in older OA patients may in part be influenced by still asymptomatic or coincident chronic heart disease. This should be addressed in future studies which include an assessment of cardiovascular outcome. 

Although the present cohort study could not consider data of healthy controls, our analysis revealed useful information to further characterize the association of sCOMP with clinical parameters in late-stage OA. In fact, for knee OA patients we identified an association between high preoperative sCOMP levels and functional impairment based on the more general FFbH score as well as the joint-specific WOMAC function sub-scale and total score. Moreover, we observed a trend for a positive association with the WOMAC pain sub-scale. Although comparable associations between the sCOMP level and specific WOMAC sub-scales as well as the WOMAC in total have been described previously in patients suffering from knee OA, the numbers of included cases were relatively small and/or the appropriate adjustment of confounders was missing [45,46,47,48].

For hip OA patients, however, no respective associations could be identified in our study, which supports the conclusion of a recent meta-analysis that COMP may have better performance as a biomarker in knee than hip OA [12]. This difference was further supported in the orientating follow-up analysis after arthroplasty in which the only statistically significant association between baseline sCOMP and the WOMAC score was found for the sub-scale stiffness in knee patients. To the best of our knowledge, this is the first report of a certain prognostic impact of preoperative sCOMP concerning early outcome after joint replacement. The otherwise missing associations argue against a major contribution of additional symptomatic OA-affected joints to preoperative sCOMP values, which confirms the lack of a statistically significant influence of bilaterality of radiologic hip or knee OA described above. 

Overall, it should be mentioned that the present findings, in particular concerning cardiac impairment, might be influenced by the pre-selected cohort for joint replacement surgery, which excludes patients at high risk for complications. Moreover, the assessment of self-reported comorbidities, although performed by physicians, might represent an additional bias to some extent. Another limitation results from the lack of an adequate control group represented by healthy individuals, which only allowed comparisons between subgroups of OA. Also, the cohorts did not comprise of patients with early stage OA. Strengths of the Ulm Osteoarthritis Study include the well-characterized study population with more than 800 patients and the availability of basal laboratory data as well as serum samples for biomarker analysis from the vast majority of participants, allowing respective multiple adjustments. 

Due to the German data protection rules we were not able to assess long-term cardiovascular mortality in relation to the identified cardiovascular marker profile in OA patients so far. It is an important future aim to identify OA patients with a higher risk for cardiovascular disease and improve respective prevention strategies.

## 5. Conclusions

Our findings provide profound evidence of an association between sCOMP and renal function. Therefore, appropriate adjustment might be recommendable for future epidemiological studies focusing on sCOMP in OA and other diseases. Besides age, gender, and BMI we found a distinct profile of markers for cardiovascular risk including NTproBNP, cystatin C, and AST associated with sCOMP values, indicating the need for more detailed investigation in correlation with the clinical manifestation of cardiovascular disease. The data further support the notion that sCOMP is more strongly associated with clinical features of advanced knee OA compared to hip OA. Moreover, sCOMP levels were rather associated with clinical parameters of OA, as assessed by the WOMAC and FFbH scores, than with the radiographic classification by Kellgren and Lawrence. Overall, this study confirmed that sCOMP is a more complex biomarker in the context of OA than previously thought.

## Figures and Tables

**Table 1:** Partial Spearman Rank Correlation Coefficients for sCOMP at baseline. Significant associations are highlighted in bold. [1] Adjusted for age, sex, and BMI. [2] Adjusted for age, sex, BMI and eGFR.

		Association to COMP [1]		Association to COMP [2]	
Parameter	Total (N = 754) Median (Q1, Q3)	Rho	*p*-Value	Rho	*p*-Value
COMP (ng/mL)	799.6 (600.4, 1068.3)				
hsCRP (mg/L)	2.5 (1.3, 5.0)	−0.065	0.074	−0.078	**0.036**
Uric Acid (µmol/L)	315.4 (267.8, 378.0)	0.044	0.264	0.023	0.559
AST (IU/L)	10.0 (8.0, 12.0)	0.128	**<0.001**	0.139	**<0.001**
ALT (IU/L)	11.0 (9.0, 16.0)	0.010	0.788	0.027	0.466
Alkaline Phosphatase (IU/L)	103.0 (85.0, 125.0)	0.076	0.055	0.071	0.076
Calcium (mmol/L)	2.4 (2.3, 2.4)	0.021	0.589	0.043	0.274
Phosphate (mmol/L)	1.0 (0.9, 1.2)	0.031	0.464	0.035	0.414
GDF-15 (ng/L)	1007.5 (780.9, 1280.5)	0.066	0.096	0.039	0.339
hs-cTnT (ng/L)	3.2 (1.5, 6.2)	0.020	0.603	0.017	0.663
hs-cTnI (ng/L)	3.9 (2.9, 5.7)	0.045	0.222	0.042	0.263
NT-proBNP (ng/L)	96.9 (51.9, 180.2)	0.102	**0.006**	0.095	**0.011**
Cystatin C (mg/L)	0.9 (0.8, 1.0)	0.171	**<0.001**	0.136	**<0.001**
Creatinine (µmol/L)	77.0 (67.0, 88.4)	0.095	**0.010**		
eGFR (mL/min/1.73 m²)	78.6 (64.9, 92.0)	−0.097	**0.009**		

**Table 2:** sCOMP levels in relation to the characteristics of patients with osteoarthritis (OA) baseline.

		sCOMP Quartile					sCOMP, ng/mL		
Parameter	Category/Statistic	Quartile 1 (N = 188)	Quartile 2 (N = 189)	Quartile 3 (N = 189)	Quartile 4 (N = 188)	Total (N = 754)	Median	*p*-Value	*p*-Value
Age (years)	Median (Q1, Q3)	61.5 (55.0, 68.0)	65.0 (57.0, 69.0)	66.0 (59.0, 71.0)	67.0 (62.0, 71.0)	65.0 (58.0, 70.0)			
	≥25 to <60	80 (42.6%)	61 (32.3%)	48 (25.4%)	32 (17.0%)	221 (29.3%)	697.95		
	≥60 to <70	69 (36.7%)	81 (42.9%)	83 (43.9%)	88 (46.8%)	321 (42.6%)	821.71		
	≥70 to <80	39 (20.7%)	47 (24.9%)	58 (30.7%)	68 (36.2%)	212 (28.1%)	885.53	<0.0001 ^a^	<0.001 ^c^
Sex	Male	60 (31.9%)	72 (38.1%)	72 (38.1%)	81 (43.1%)	285 (37.8%)	815.13		
	Female	128 (68.1%)	117 (61.9%)	117 (61.9%)	107 (56.9%)	469 (62.2%)	779.53	0.047 ^a^	<0.001 ^c^
BMI (kg/m²)	Median (Q1, Q3)	27.1 (25.0, 29.5)	28.0 (25.7, 30.8)	28.1 (25.6, 31.1)	28.3 (25.7, 32.0)	27.8 (25.4, 30.8)			
	≥0 to <25	48 (25.5%)	40 (21.2%)	43 (22.8%)	39 (20.7%)	170 (22.5%)	787.03		
	≥25 to <30	99 (52.7%)	93 (49.2%)	84 (44.4%)	78 (41.5%)	354 (46.9%)	759.79		
	≥30 to <35	33 (17.6%)	47 (24.9%)	45 (23.8%)	54 (28.7%)	179 (23.7%)	830.58		
	≥35	8 (4.3%)	9 (4.8%)	17 (9.0%)	17 (9.0%)	51 (6.8%)	905.45	0.002 ^a^	0.005 ^c^
Localization of OA	Hip	109 (58.0%)	104 (55.0%)	101 (53.4%)	80 (42.6%)	394 (52.3%)	777.75		
	Knee	79 (42.0%)	85 (45.0%)	88 (46.6%)	108 (57.4%)	360 (47.7%)	839.53	0.338 ^b^	0.487 ^d^
Laterality of OA	Unilateral OA	30 (16.0%)	32 (16.9%)	21 (11.1%)	22 (11.7%)	105 (13.9%)	718.09		
	Bilateral OA	143 (76.1%)	141 (74.6%)	154 (81.5%)	144 (76.6%)	582 (77.2%)	806.22	0.443 ^b^	0.426 ^d^
	Unknown	15 (8.0%)	16 (8.5%)	14 (7.4%)	22 (11.7%)	67 (8.9%)			
Generalization of OA	Not-generalized OA	118 (62.8%)	116 (61.4%)	106 (56.1%)	101 (53.7%)	441 (58.5%)	781.15		
	Generalized OA	28 (14.9%)	39 (20.6%)	44 (23.3%)	49 (26.1%)	160 (21.2%)	875.00	0.110 ^b^	0.063 ^d^
	Unknown	42 (22.3%)	34 (18.0%)	39 (20.6%)	38 (20.2%)	153 (20.3%)			
Cause of OA	Primary OA	108 (57.4%)	124 (65.6%)	117 (61.9%)	108 (57.4%)	457 (60.6%)	792.86		
	Secondary OA	75 (39.9%)	59 (31.2%)	68 (36.0%)	72 (38.3%)	274 (36.3%)	815.03	0.353 ^b^	0.417 ^d^
	Unknown	5 (2.7%)	6 (3.2%)	4 (2.1%)	8 (4.3%)	23 (3.1%)			
Smoking Status	Never	115 (61.2%)	110 (58.2%)	103 (54.5%)	109 (58.0%)	437 (58.0%)	784.81		
	Former	48 (25.5%)	62 (32.8%)	57 (30.2%)	54 (28.7%)	221 (29.3%)	800.03		
	Current	25 (13.3%)	17 (9.0%)	29 (15.3%)	25 (13.3%)	96 (12.7%)	822.34	0.192 ^b^	0.210 ^d^
**Comorbidities**									
Diabetes	No	178 (94.7%)	176 (93.1%)	166 (87.8%)	169 (89.9%)	689 (91.4%)	787.22		
	Yes	10 (5.3%)	13 (6.9%)	22 (11.6%)	19 (10.1%)	64 (8.5%)	857.81	0.446 ^b^	0.741 ^d^
	Unknown	0 (0.0%)	0 (0.0%)	1 (0.5%)	0 (0.0%)	1 (0.1%)			
Hypertension	No	93 (49.5%)	105 (55.6%)	95 (50.3%)	78 (41.5%)	371 (49.2%)	772.54		
	Yes	95 (50.5%)	84 (44.4%)	94 (49.7%)	110 (58.5%)	383 (50.8%)	818.21	0.918 ^b^	0.852 ^d^
Cardiac	No	181 (96.3%)	180 (95.2%)	181 (95.8%)	180 (95.7%)	722 (95.8%)	799.56		
Infarction	Yes	7 (3.7%)	9 (4.8%)	7 (3.7%)	8 (4.3%)	31 (4.1%)	776.07	0.668 ^b^	0.538 ^d^
	Unknown	0 (0.0%)	0 (0.0%)	1 (0.5%)	0 (0.0%)	1 (0.1%)			
Cardiac	No	161 (85.6%)	159 (84.1%)	152 (80.4%)	138 (73.4%)	610 (80.9%)	785.20		
Insufficiency	Yes	27 (14.4%)	30 (15.9%)	36 (19.0%)	50 (26.6%)	143 (19.0%)	877.47	0.382 ^b^	0.525 ^d^
	Unknown	0 (0.0%)	0 (0.0%)	1 (0.5%)	0 (0.0%)	1 (0.1%)			

(Q1, Q3) interquartile range; N = number of patients. Multiple linear regression models: ^a^ not adjusted; ^b^ adjusted for age, sex and BMI; ^c^ adjusted for eGFR; ^d^ adjusted for age, sex, BMI and eGFR. Significant associations are highlighted in bold.

**Table 3:** FFbH Hannover Functionality Status of patients with knee OA. Baseline and follow-up six months after surgery. Multiple linear regression model adjusted for age, sex, BMI, and eGFR. Significant associations are highlighted in bold.

	Baseline			Follow-Up 6 Months		
Predictors	β-Coefficients	CI	*p*-Value	β-Coefficients	CI	*p*-Value
ln (COMP)	−3.87	−7.60–−0.15	**0.042**	−1.46	−5.34–2.43	0.463
Age	−0.35	−0.64–−0.07	**0.016**	−0.27	−0.59–0.05	0.098
Sex: Female	−7.07	−11.07–−3.07	**0.001**	−5.63	−9.79–−1.47	**0.009**
BMI	−0.30	−0.73–0.12	0.164	−0.46	−0.91–−0.01	**0.048**
eGFR	−0.07	−0.17–0.04	0.199	−0.08	−0.19–0.03	0.162
Observations	297			236		
R^2^/adjusted R^2^	0.096/0.080			0.067/0.046		

**Table 4:** WOMAC Scores of patients with knee OA. (A) Baseline and (B) follow-up six months after surgery. Multiple linear regression models adjusted for age, sex, BMI, and eGFR. Significant associations are highlighted in bold.

(**A**) **WOMAC Scores—Baseline; Knee OA Patients.**												
	**Pain Score**			**Stiffness Score**			**Function Score**			**Total Score**		
Predictors	β-coefficients	CI	*p*	β-coefficients	CI	*p*	β-coefficients	CI	*p*	β-coefficients	CI	*p*
ln (COMP)	0.72	−0.06–1.50	0.071	0.40	−0.15–0.94	0.154	3.56	0.83–6.29	**0.011**	4.42	0.87–7.97	**0.015**
Age	−0.07	−0.13–−0.01	**0.031**	−0.02	−0.06–0.02	0.311	−0.12	−0.32–0.09	0.270	−0.18	−0.45–0.09	0.184
Sex: Female	2.25	1.38–3.11	**<0.001**	0.82	0.22–1.43	**0.008**	7.02	3.97–10.06	**<0.001**	9.12	5.17–13.07	**<0.001**
BMI	0.02	−0.07–0.11	0.700	0.00	−0.06–0.07	0.911	0.05	−0.26–0.36	0.747	0.02	−0.38–0.42	0.931
eGFR	0.01	−0.01–0.03	0.418	0.01	−0.01–0.02	0.495	0.04	−0.04–0.11	0.338	0.04	−0.06–0.14	0.478
Observations	278			278			262			278		
R^2^/adjusted R^2^	0.097/0.080			0.031/0.013			0.088/0.070			0.082/0.065		
(**B**) **WOMAC Scores—Follow-up Six Months; Knee OA Patients.**												
	**Pain Score**			**Stiffness Score**			**Function Score**			**Total Score**		
Predictors	β-coefficients	CI	*p*	β-coefficients	CI	*p*	β-coefficients	CI	*p*	β-coefficients	CI	*p*
ln (COMP)	0.93	−0.20–2.06	0.109	0.53	0.03–1.02	**0.037**	1.98	−1.74–5.70	0.297	3.10	−2.09–8.30	0.243
Age	−0.04	−0.13–0.05	0.379	−0.04	−0.09–−0.00	**0.029**	0.10	−0.20–0.39	0.523	0.03	−0.38–0.45	0.879
Sex: Female	0.75	−0.45–1.95	0.222	0.11	−0.41–0.64	0.673	-0.06	−4.07–3.96	0.977	0.42	−5.17–6.01	0.884
BMI	0.07	−0.06–0.19	0.283	0.02	−0.04–0.07	0.507	0.48	0.07–0.89	**0.021**	0.67	0.10–1.23	**0.021**
eGFR	0.03	−0.00–0.06	0.091	0.01	−0.01–0.02	0.233	0.12	0.00–0.23	**0.042**	0.14	−0.01–0.30	0.072
Observations	242			253			214			208		
R^2^/adjusted R^2^	0.034/0.014			0.045/0.026			0.052/0.029			0.050/0.027		

## Supplementary Materials

The following are available online at https://www.mdpi.com/2077-0383/9/1/268/s1, Figure S1: Trajectories of the FFbH Hannover Functionality Status of patients with hip OA. Figure S2: Trajectories of separated WOMAC sub-scales pain (A), stiffness (B), and function (C), and total WOMAC (D) of patients with hip OA at baseline and six months follow-up. Table S1A,B: Kellgren and Lawrence scores. Table S2: FFbH Hannover Functionality Status of patients with knee OA; baseline and follow-up six months after surgery; adjusted for age, sex, BMI, and eGFR. Table S3A,B: WOMAC Scores of patients with hip OA; (A) baseline and (B) follow-up six months after surgery; adjusted for age, sex, BMI, and eGFR.

## Appendix A

**Table A1:** Descriptive FFbH (hip and knee OA) at baseline given as mean (SD). Multiple linear regression model adjusted for age, sex, BMI, and eGFR.

		COMP Quarter				
Parameter	Category/Statistic	Quarter 1 (N = 188)	Quarter 2 (N = 189)	Quarter 3 (N = 189)	Quarter 4 (N = 188)	Total (N = 754)
FFbH Functional	Baseline	67.8 (15.0)	67.1 (15.2)	68.2 (15.7)	66.2 (16.4)	67.4 (15.5)
Capacity (%)	6 months	80.4 (14.1)	82.5 (14.9)	78.8 (15.7)	79.1 (15.7)	80.1 (15.2)

**Table A2:** Descriptive WOMAC Score (hip and knee OA) at baseline given as mean (SD). Multiple linear regression model adjusted for age, sex, BMI, and eGFR.

		COMP Quarter				
Parameter	Category/Statistic	Quarter 1 (N = 188)	Quarter 2 (N = 189)	Quarter 3 (N = 189)	Quarter 4 (N = 188)	Total (N = 754)
WOMAC Pain	Baseline	12.0 (3.4)	11.8 (3.3)	11.4 (3.5)	12.0 (3.5)	11.8 (3.4)
	6 months	4.7 (4.0)	4.6 (3.7)	5.0 (4.4)	5.1 (4.2)	4.8 (4.1)
WOMAC Stiffness	Baseline	4.6 (2.1)	4.7 (2.0)	4.5 (2.2)	4.7 (2.0)	4.6 (2.1)
	6 months	2.4 (1.7)	2.4 (1.8)	2.9 (2.0)	2.7 (1.8)	2.6 (1.8)
WOMAC Function	Baseline	38.2 (11.0)	39.7 (10.9)	38.4 (11.9)	38.3 (11.8)	38.7 (11.2)
	6 months	18.5 (12.5)	16.9 (13.3)	20.1 (14.0)	19.0 (14.6)	18.6 (13.6)
WOMAC Total	Baseline	54.8 (14.7)	56.1 (14.4)	54.3 (14.7)	54.9 (15.3)	55.0 (14.8)
	6 months	25.9 (17.4)	23.7 (17.9)	28.5 (19.4)	26.9 (19.4)	26.2 (18.5)
