# Supplementary material for: Serum Cartilage Oligomeric Matrix Protein in Late-Stage Osteoarthritis: Association with Clinical Features, Renal Function, and Cardiovascular Biomarkers
Source: J Clin Med. 2020 Jan 18;9(1):268. doi: 10.3390/jcm9010268 (PMC7019234; doi:10.3390/jcm9010268)
Supplement: Supplementary file 1 [file jcm-09-00268-s001.zip › Riegger-COMP-Revision-Supplemental Material/Figure S2.docx]

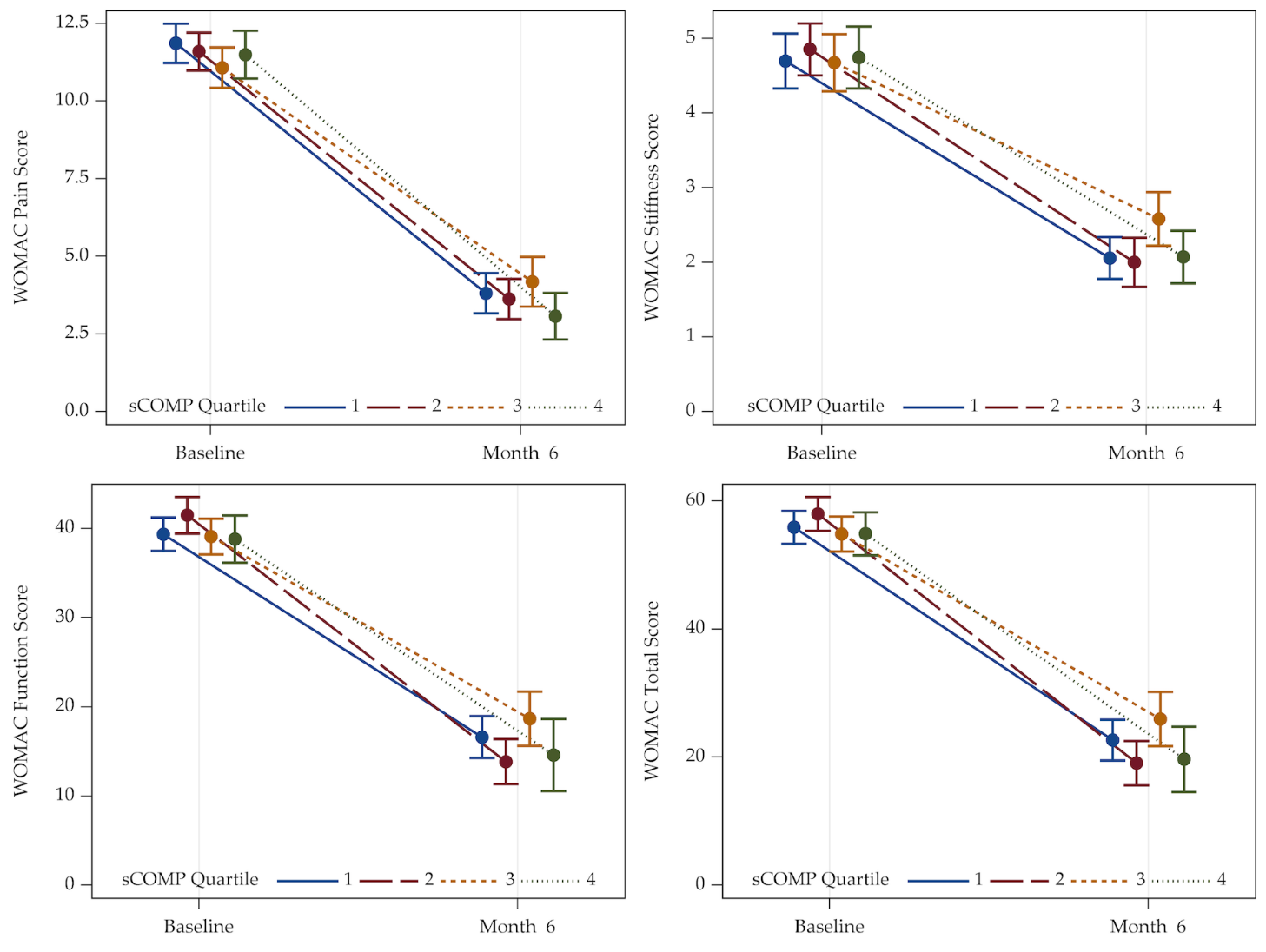


**Figure S1**. Figure 2. Trajectories of separated WOMAC sub-scales pain (A), stiffness (B) and function (C) and total WOMAC scores (D) of patients with hip OA at baseline and six months follow-up. Higher values reflect more severe impairment. Values are given as means with two-sided 95% confidence limits. WOMAC=Western Ontario and McMaster University Osteoarthritis Index; Q= quartile.
