# Supplementary material for: Serum Cartilage Oligomeric Matrix Protein in Late-Stage Osteoarthritis: Association with Clinical Features, Renal Function, and Cardiovascular Biomarkers
Source: J Clin Med. 2020 Jan 18;9(1):268. doi: 10.3390/jcm9010268 (PMC7019234; doi:10.3390/jcm9010268)
Supplement: Supplementary file 1 [file jcm-09-00268-s001.zip › Riegger-COMP-Revision-Supplemental Material/Table S1A+B.docx]

**Table S1:** Kellgren & Lawrence Score oft he **(A)** hip and **(B)** knee patients included in the study. Multiple linear regression model [1] Adjusted for age, sex, and BMI. [2] Adjusted for age, sex, BMI, and eGFR.

**A)** Patients with hip OA (total: N=394).

| **Score** | **Number of patients** | **Median COMP, ng/mL** | **p-value[1]** | **p-value[2]** |
| --- | --- | --- | --- | --- |
| 2 | 5 (1.3%) | 752.56 |  |  |
| 3 | 83 (21.1%) | 735.44 |  |  |
| 4 | 290 (73.6%) | 780.34 | 0.414 | 0.482 |
| Missing | 16 (4.1 %) |  |  |  |

**B)** Patients with knee OA (total: N=360).

| **Score** | **Number of patients** | **Median COMP, ng/mL** | **p-value[1]** | **p-value[2]** |
| --- | --- | --- | --- | --- |
| 2 | 7 (1.9%) | 756.64 |  |  |
| 3 | 169 (46.9%) | 877.11 |  |  |
| 4 | 171 (47.5%) | 835.44 | 0.888 | 0.942 |
| Missing | 13 (3.6%) |  |  |  |
