# Supplementary material for: Serum Cartilage Oligomeric Matrix Protein in Late-Stage Osteoarthritis: Association with Clinical Features, Renal Function, and Cardiovascular Biomarkers
Source: J Clin Med. 2020 Jan 18;9(1):268. doi: 10.3390/jcm9010268 (PMC7019234; doi:10.3390/jcm9010268)
Supplement: Supplementary file 1 [file jcm-09-00268-s001.zip › Riegger-COMP-Revision-Supplemental Material/Table S2.docx]

**Table S2:** FFbH Hannover Functionality Status of patients with hip OA. Baseline and follow-up six months after surgery. Multiple linear regression model adjusted for age, sex, BMI, and eGFR.

|  | **Baseline** | | | **FU 6 Months** | | |
| --- | --- | --- | --- | --- | --- | --- |
| Predictors | β- coefficients | CI | p | β- coefficients | CI | p |
| ln(COMP) | -1.45 | -5.01 – 2.12 | 0.427 | -2.69 | -6.94 – 1.56 | 0.216 |
| Age | -0.20 | -0.39 – -0.01 | **0.038** | -0.08 | -0.30 – 0.14 | 0.465 |
| Sex: Female | -8.33 | -11.65 – -5.01 | **<0.001** | -5.03 | -8.83 – -1.23 | **0.010** |
| BMI | -0.54 | -0.92 – -0.16 | **0.006** | -0.58 | -1.03 – -0.13 | **0.012** |
| eGFR | -0.03 | -0.13 – 0.07 | 0.577 | 0.03 | -0.09 – 0.16 | 0.623 |
| Observations | 327 | | | 263 | | |
| R^2^ / adjusted R^2^ | 0.114 / 0.100 | | | 0.069 / 0.051 | | |
