# Supplementary material for: Serum Cartilage Oligomeric Matrix Protein in Late-Stage Osteoarthritis: Association with Clinical Features, Renal Function, and Cardiovascular Biomarkers
Source: J Clin Med. 2020 Jan 18;9(1):268. doi: 10.3390/jcm9010268 (PMC7019234; doi:10.3390/jcm9010268)
Supplement: Supplementary file 1 [file jcm-09-00268-s001.zip › Riegger-COMP-Revision-Supplemental Material/Table S3A+B.docx]

**Table S3A+B:** WOMAC Scores of patients with hip OA. (A) Baseline and (B) follow-up six months after surgery. Multiple linear regression models adjusted for age, sex, BMI, and eGFR.

|  | **Pain Score** | | | **Stiffness Score** | | | **Function Score** | | | **Total Score** | | |
| --- | --- | --- | --- | --- | --- | --- | --- | --- | --- | --- | --- | --- |
| Predictors | β-coefficients | CI | p | β-coefficients | CI | p | Β-coefficients | CI | p | β-coefficients | CI | p |
| ln(COMP) | 0.15 | -0.73 – 1.02 | 0.743 | 0.26 | -0.27 – 0.78 | 0.340 | 0.27 | -2.52 – 3.07 | 0.848 | 0.76 | -3.03 – 4.54 | 0.696 |
| Age | 0.01 | -0.04 – 0.05 | 0.699 | -0.03 | -0.06 – -0.00 | **0.030** | -0.04 | -0.19 – 0.10 | 0.547 | -0.07 | -0.26 – 0.13 | 0.501 |
| Sex: Female | 1.89 | 1.08 – 2.70 | **<0.001** | 0.32 | -0.16 – 0.79 | 0.188 | 5.31 | 2.74 – 7.89 | **<0.001** | 7.35 | 3.92 – 10.78 | **<0.001** |
| BMI | 0.11 | 0.02 – 0.21 | **0.021** | 0.04 | -0.01 – 0.10 | 0.111 | 0.40 | 0.11 – 0.70 | **0.008** | 0.53 | 0.14 – 0.93 | **0.008** |
| eGFR | 0.01 | -0.02 – 0.03 | 0.518 | 0.00 | -0.01 – 0.02 | 0.658 | -0.01 | -0.09 – 0.07 | 0.793 | 0.00 | -0.10 – 0.11 | 0.966 |
| Observations | 312 | | | 315 | | | 309 | | | 314 | | |
| R^2^ / adjusted R^2^ | 0.081 / 0.066 | | | 0.033 / 0.017 | | | 0.075 / 0.059 | | | 0.076 / 0.061 | | |

**B)** WOMAC scores – follow-up six months; hip OA patients.

|  | **Pain Score** | | | **Stiffness Score** | | | **Function Score** | | | **Total Score** | | |
| --- | --- | --- | --- | --- | --- | --- | --- | --- | --- | --- | --- | --- |
| Predictors | β-coefficients | CI | p | β-coefficients | CI | p | β-coefficients | CI | p | β-coefficients | CI | p |
| ln(COMP) | -0.23 | -1.23 – 0.76 | 0.646 | 0.27 | -0.19 – 0.72 | 0.250 | 0.25 | -3.70 – 4.20 | 0.902 | 0.32 | -5.10 – 5.74 | 0.909 |
| Age | -0.02 | -0.07 – 0.04 | 0.548 | -0.01 | -0.04 – 0.01 | 0.361 | -0.04 | -0.25 – 0.17 | 0.681 | -0.11 | -0.39 – 0.17 | 0.455 |
| Sex: Female | -0.03 | -0.93 – 0.87 | 0.948 | 0.08 | -0.33 – 0.50 | 0.692 | 1.11 | -2.50 – 4.71 | 0.548 | 1.42 | -3.43 – 6.26 | 0.568 |
| BMI | 0.01 | -0.09 – 0.12 | 0.798 | 0.05 | 0.00 – 0.10 | **0.049** | 0.50 | 0.09 – 0.91 | **0.017** | 0.56 | 0.01 – 1.12 | **0.048** |
| eGFR | -0.02 | -0.05 – 0.01 | 0.146 | -0.01 | -0.02 – 0.01 | 0.407 | 0.02 | -0.09 – 0.14 | 0.688 | -0.01 | -0.17 – 0.15 | 0.899 |
| Observations | 282 | | | 285 | | | 238 | | | 230 | | |
| R^2^ / adjusted R^2^ | 0.009 / -0.009 | | | 0.024 / 0.007 | | | 0.028 / 0.007 | | | 0.021 / -0.001 | | |
